# Polymer-Ceramic Spiral Structured Scaffolds for Bone Tissue Engineering: Effect of Hydroxyapatite Composition on Human Fetal Osteoblasts

**DOI:** 10.1371/journal.pone.0085871

**Published:** 2014-01-27

**Authors:** Xiaojun Zhang, Wei Chang, Paul Lee, Yuhao Wang, Min Yang, Jun Li, Sangamesh G. Kumbar, Xiaojun Yu

**Affiliations:** 1 Department of Chemistry, Chemical Biology, and Biomedical Engineering, Stevens Institute of Technology, Hoboken, New Jersey, United States of America; 2 Department of Physics and Mathematics, School of Biomedical Engineering, Fourth Military Medical University, Xi'an, Shaanxi, People's Republic of China; 3 Department of Orthopaedic Surgery, University of Connecticut Health Center, Farmington, Connecticut, United States of America; Instituto de Engenharia Biomédica, University of Porto, Portugal

## Abstract

For successful bone tissue engineering, a scaffold needs to be osteoconductive, porous, and biodegradable, thus able to support attachment and proliferation of bone cells and guide bone formation. Recently, hydroxyapatites (HA), a major inorganic component of natural bone, and biodegrade polymers have drawn much attention as bone scaffolds. The present study was designed to investigate whether the bone regenerative properties of nano-HA/polycaprolactone (PCL) spiral scaffolds are augmented in an HA dose dependent manner, thereby establishing a suitable composition as a bone formation material. Nano-HA/PCL spiral scaffolds were prepared with different weight ratios of HA and PCL, while porosity was introduced by a modified salt leaching technique. Human fetal osteoblasts (hFOBs) were cultured on the nano-HA/PCL spiral scaffolds up to 14 days. Cellular responses in terms of cell adhesion, viability, proliferation, differentiation, and the expression of bone-related genes were investigated. These scaffolds supported hFOBs adhesion, viability and proliferation. Cell proliferation trend was quite similar on polymer-ceramic and neat polymer spiral scaffolds on days 1, 7, and 14. However, the significantly increased amount of alkaline phosphatase (ALP) activity and mineralized matrix synthesis was evident on the nano-HA/PCL spiral scaffolds. The HA composition in the scaffolds showed a significant effect on ALP and mineralization. Bone phenotypic markers such as bone sialoprotein (BSP), osteonectin (ON), osteocalcin (OC), and type I collagen (Col-1) were semi-quantitatively estimated by reverse transcriptase polymerase chain reaction analysis. All of these results suggested the osteoconductive characteristics of HA/PCL nanocomposite and cell maturation were HA dose dependent. For instance, HA∶PCL = 1∶4 group showed significantly higher ALP mineralization and elevated levels of BSP, ON, OC and Col-I expression as compared other lower or higher ceramic ratios. Amongst the different nano-HA/PCL spiral scaffolds, the 1∶4 weight ratio of HA and PCL is shown to be the most optimal composition for bone tissue regeneration.

## Introduction

Bone defects caused by trauma, tumor resection, pathological degeneration, or congenital deformity are one of the majors clinical challenges in orthopedic treatments [1]. Every year, there are around 2.2 million bone graft surgeries in the worldwide [2], [3], and this number is increasing annually [4]. Currently, autologous bone grafts remains the gold standard in the treatment of bone defects [5], [6]. However, significant issues of autografts such as the need for two surgical procedures for harvesting and implantation, limited supply, and donor site morbidity [7]. Bone allografts, alternatives to autografts, suffer from drawbacks such as cost-expensive, disease transmission, and adverse host immune reaction [4]. These significant caveats have increased the need for synthetic bone graft substitutes to repair bone defects.

Hydroxyapatite (HA) is the major inorganic component of natural bone and has long been used as an orthopedic and dental material [8], [9]. HA is well known for its excellent biocompatibility, osteoconductive potential, slow degradation, non-cytotoxicicity, non-inflammatory, as well as non-immunogenic properties [10], [11]. Although HA is a constituent of the natural bone, the intrinsic properties of HA, such as difficulty in remodeling, hardness, fragility, and lack of flexibility, make it difficult to be shaped in the specific form required for bone repair and implantation, which limits its application as a load-bearing implant scaffold material [12], [13]. To circumvent these drawbacks great efforts are focused on combining HA with polymers, which may not only overcome the mechanical properties of HA but also improve the osteoconductivity properties of the polymers. Some nature polymers, such as collagen [14], [15], chitosan [16], chitin [17], alginate [18], and silk [19] are employed for combination with HA to apply in bone tissue engineering. But the problem of using such natural materials is their inferiority in mechanical properties [20]. Therefore, many studies have been reported on combination HA with biodegradable synthetic materials, such as poly (lactic acid) [21], poly (ε-caprolactone) (PCL) [22]–[24], poly (lactic-co-glycolic acid) [20], Poly (D,L-lactide) [25], to circumvent the drawbacks of natural polymers.

Among the available biodegradable synthetic polymers, PCL polymer-based composites have been focused with more attention than other synthetic polymer composite for bone tissue engineering applications, due to its sustained biodegradability, elastic characteristics, and low inflammatory response [26]. Many studies had focused on the mechanical characters of HA/PCL scaffold as a bone graft substitute. Yu, et al. evaluated the microstructures and mechanical properties of HA/PCL scaffold [27]. Johari, et al. also investigated the mechanical properties of nano-fluoridated HA/PCL scaffolds in order to obtain an optimized composition [28]. While Eosoly, et al. fabricated PCL/HA composites and examined the effect of HA addition on surface roughness, wettability, mechanical behavior and surface morphology and MC-3T3 osteoblast like cells' activity [29]. In regards to the cell responses of different studies, there is conflicting data reported in literature about the effect of HA addition to PCL composites in terms of cell attachment, proliferation and differentiation. Some authors demonstrated that PCL/HA scaffolds improved osteoblasts differentiation and matrix mineralization [30]. However, Chim et al. showed that the presence of HA in PCL scaffolds has little or no effect on biological response [31]. Furthermore, Causa et al. found that human osteoblasts cultured on different volume ratio HA/PCL scaffolds had similar cell viability, but decreased cell differentiation with increasing the volume of HA in the HA/PCL scaffolds [32].

It is still unclear whether HA/PCL scaffold stimulates its own osteogenic capability in an HA dose dependent manner *in vitro* and whether the ratio of HA and PCL is optimized in previous HA/PCL scaffolds with regard to osteoconductive characteristics as application in bone tissue engineering. Our group has designed a spiral structured scaffold which can provide thinner scaffold walls for cells to easily grow across and thorough gaps between scaffold walls to ensure sufficient nutrient supply and metabolic waste removal [33], [34]. In the present study, spiral scaffolds of various nano-HA/PCL composites were prepared with different weight ratios of HA and PCL. The morphological structures of these spiral scaffolds were analyzed. Moreover, human fetal osteoblasts (hFOBs) cellular responses to the composites were examined in terms of the cellular attachment, proliferation, differentiation, and mineralized matrix deposition as well as the expression of bone-related genes. The aim of this study was designed to establish a suitable composition of nano-HA/PCL as a scaffold of bone graft substitute for clinical applications such as non union bone defect repairs.

## Materials and Methods

### 1. Preparation and characterization of nano-HA/PCL spiral scaffolds

The scaffolds were prepared by a salt leaching method with minor modifications. Briefly, PCL (Sigma-Aldrich, USA) was dissolved in Dichloromethane (DCM) with a ratio of 8% (w/v) by stirring the solution for overnight. Nanosized HA (particle size <200 nm, Sigma-Aldrich, USA) were added to PCL/DCM solution at 1∶8, 1∶4, and 1∶2 of the PCL weight ratios. The resultant mixture was stirred for 24 h at room temperature to increase homogenization. PCL/DCM solution without the addition of nano-HA powder was used as a control. After coating and drying a monolayer of sodium chloride (crystal, Fisher Scientific, USA) particles, 250–400 µm in size, on the bottom of glass petri dish, 7.5 ml of PCL or nano-HA/PCL solutions were added to the glass petri dish. Uniformity was ensured by moving the petri dish around until the polymer solution was evenly distributed. Afterwards DCM was allowed to evaporate in the hood for 3 to 5 min. Subsequently, the same sized sodium chloride was added to the surface of PCL or nano-HA/PCL solution to form another salt layer, which was pressed after 2 min. Then, DCM was evaporated under reduced pressure to form a dry layer. Once all DCM is evaporated, overnight salt leaching in DI water, the highly porous nano-HA/PCL or PCL sheets were obtained with a thickness of 0.3–0.4 mm. Each sheet was cut into rectangular strips that measure 45 mm×5 mm. Each strip was then rolled into a spiral shape and held in place with a strip of copper sheeting, which acts as the mold to form a spiral structure. After being incubated at 45°C in an oven for 30 min, the scaffold was immediately transferred to −80°C for 24 h to immobilize the shape. The copper mold was finally removed. Both stereomicroscopy (Nikon 1500z stereo optical microscope, Japanese) and scanning electron microscopy (SEM, Leo 982 FEG-SEM, Zeiss, Germany) were used to analyze the morphology, the porous structure, and the integration of the scaffolds.

### 2. Cell culture and seeding

HFOB cells (hFOB 1.19, ATCC CRL-11372, USA) were cultured in DMEM (Gibco, USA) supplemented with 10% fetal calf serum and 1% penicillin/streptomycin in a humidified incubator at 37°C with 5% CO_2_. The medium was changed every other day.

The scaffolds were sterilized with 70% ethanol for 1 h and then irradiated by UV light for 30 min in PBS. Following rinsing with PBS 3 times, the scaffolds were soaked in cell culture medium for overnight in incubator. In order to uniformly seed cells onto the scaffolds, hFOBs were added into 15 ml tube, which was contained with the spiral scaffolds, at the cell density of 1×10^5^ cells per each scaffold and cultured on the rotating shaker at 30 rpm in the humidified incubator at 37°C with 5% CO_2_. After 2 h, cell–scaffold constructs were removed from the tube and transferred into 24-well tissue culture plates containing 1 ml of complete media. The medium was changed every day, and the cultures were maintained for 21 days. At the indicated time endpoints, cell–scaffold constructs were removed and characterized for cell viability, proliferation, differentiation, mineralized matrix synthesis, and bone related gene expression, respectively.

### 3. Cell viability

To determine cell viability within the scaffolds, cell–scaffold constructs were stained using the LIVE/DEAD® Viability/Cytotoxicity kit for mammalian cells (Invitrogen Life Technologies, USA), according to manufacturer's instructions. Briefly, after 7 days culture, small sections cut from the cell-seeded scaffolds were incubated with 100 µl of the Live/Dead solution containing 4 µM ethidium homodimer-1 (EthD-1) and 2 µM calcein AM at the room temperature for 30 min, then mounted onto a glass slide using SlowFade® Gold Antifade Reagent with DAPI (Invitrogen Life Technologies, USA), and viewed with confocal laser microscopy (Zeiss LSM5 Pascal, Germany) with 494 nm (green, Calcein) and 528 nm (red, EthD-1) excitation filters. Images were captured using Zeiss LSM Data Server software. In order to quantitatively analyze, five randomly chosen areas from each sample were captured and the green areas and red areas of each image were recorded.

### 4. Cell morphology

The 4 day incubation period, cell–scaffold constructs were fixed by 4% paraformaldehyde at 4°C for overnight. Some samples were permeabilized with 0.1% Triton X-100 for 15 min, and then blocked with 1% bovine serum albumin for 30 min. Following that the F-actin cytoskeleton of osteoblasts was stained with Rhodamine Phalloidin (Invitrogen Life Technologies, USA), and the nucleus was stained with DAPI (Invitrogen Life Technologies, USA). The other samples were embedded in paraffin and sectioned at a thickness of 5 µm and then stained with hematoxylin and eosin (H&E).

### 5. Cell proliferation

Cell proliferation was assessed using the MTS assay (CellTiter96™ AQueous Assay, Promega, USA) and PicoGreen DNA quantification assay (Quant-iT™ PicoGreen® dsDNA Assay Kit, Invitrogen Life Technologies, USA). For MTS assay, cell–scaffold constructs were transferred to new cell culture plates after 1, 7, and 14 days culture. One hundred microliter of pre-warmed MTS solution with 1 ml culture medium was added to each well with continuous culture for 3 h. Two hundred microliter of supernatant from each well was then transferred to a 96-well plate and the absorbance was measured at 490 nm using a SYNERGY HT plate reader (BIO-TEK, USA). Six specimens for each group were tested, and each test was repeated three times.

For PicoGreen DNA quantification assay, cell–scaffold constructs were moved to new 48-well plates, washed with PBS for three times, and treated with 500 µl TE buffer (10 mM Tris-HCl, 1 mM EDTA, pH 7.5) at the indicated time endpoints. Following three freeze–thaw cycles, a volume of 100 µl of supernatant was taken from the samples and added into 100 µl of PicoGreen reagent (diluted 1∶200 in TE buffer). Samples were incubated in darkness for 5 min before fluorescence reading at the excitation and emission wavelengths of 485 nm and 520 nm, respectively. To minimize photobleaching, the time used for fluorescence measurement was kept constant for all samples.

### 6. ALP activity

ALP activity of the cells was measured as an early marker of the maintenance of the osteoblastic phenotype using EnzoLyte pNPP Alkaline Phosphatase Assay Kit (AnaSpec, San Jose, USA) according to the manufacturer's protocol. Briefly, cell–scaffold constructs were homogenized in 500 µl lysis buffer provided in the kit. Lysate was centrifuged for 15 min at 10,000 g at 4°C. Following that, 50 µl supernatant was added to 50 µl of pNPP ALP substrate solution and incubated at 37°C for 60 min. The reaction was then stopped by adding 50 µl of stop solution into each well. The activity of ALP in cell lysates was measured with a microplate reader (SYNERGY HT, BioTek, USA) at 405 nm. The results were normalized into total cellular protein, which was measured using a Quickstart Bradford Protein assay kit (Bio-Rad, USA) [35]. In short, 150 µl of cell lysate was mixed with 150 µl of working reagent and incubated for 5 min at room temperature. The resulting optical density was read at 595 nm. Six specimens for each group were tested, and each test was repeated three times.

### 7. Calcium expression

Mineralized matrix synthesis at days 21 was analyzed using an Alizarin Red staining method for calcium deposition [36]. Cell-scaffold constructs were fixed by 4% paraformaldehyde at 4°C for overnight and subsequently stained with 2% Alizarin Red (Sigma-Aldrich, USA) solution for 10 min. To quantify the calcium amount on the scaffold, after washed with acetone and xylene, the red matrix precipitate was solublized in 10% cetylpyridinium chloride (Sigma-Aldrich, USA), and the optical density was read at 562 nm. Nano-HA/PCL spiral scaffolds without seeding hFOBs were served as the blank control. Six specimens for each group were tested, and each test was repeated three times.

### 8. RNA extraction and RT-PCR assay

The expression of osteogenic genes in cell-scaffold constructs were examined by reverse transcription-polymerase chain reaction (RT-PCR). Total RNA was extracted with commercial Trizol Reagent (Invitrogen Life Technologies, USA) from nano-HA/PCL or PCL spiral scaffolds seeded with hFOBs for 21 days. One microgram of total RNA was reverse transcribed with reverse transcriptase (Promega, USA) according to the manufacturer's instructions. All PCR experiments were performed with Taq polymerase (Promega, USA). The primers used as follows: bone sialoprotein (BSP; sense, 5′-AATGAAAACGAAGAAAGCGAAG-3′; antisense, 5′-ATCATAGCCATCGTAGCCTTGT-3′; 450 bp), osteonectin (ON; sense, 5′-TGGATCTTCTTTCTCCTTT-3′; antisense, 5′-TTCTGCTTCTCAGTCAGA-3′; 569 bp), ALP (sense, 5′-TGGAGCTTCAGAAGCTCAACACCA-3′; antisense, 5′-ATCTCGTTGTCTGAGTACCAGTCC-3′; 454 bp), osteocalcin (OC; sense, 5′-ATGAGAGCCCTCACACTCCTC-3′; antisense, 5′-GCCGTAGAAGCGCCGATAGGC-3′; 294 bp), type I collagen (Col-1; sense, 5′-GGACACAATGGATTGCAAGG-3′; antisense, 5′-TAACCACTGCTCCACTCTGG-3′; 461 bp), and β-actin (sense, 5′-GGCATCGTGATGGACTCCG-3′; antisense, 5′-GCTGGAAGGTGGACAGCGA-3′; 613 bp) served as the house-keeping gene control. The cycling conditions consisted of the initial denaturation at 95°C for 5 min, followed by 35 cycles of denaturing at 95°C for 30 s, annealing at 55°C for 1 min, and polymerization at 72°C for 1 min, and then a final 10 min extension at 72°C. The PCR products were separated and visualized on 1.5% agarose gel containing 5 g/L ethidium bromide. In addition, band intensity was normalized to that of β-actin.

### 9. Statistics

Quantitative data were reported as mean ± standard deviation. The overall statistical significance of any difference resulting from treatment was determined by analysis of variance (ANOVA). If significance was observed, the Student's t test was used to test for differences between group means. A value of *p*<0.05 was considered to be statistically significant.

## Results

### 1. Characterization of nano-HA/PCL spiral scaffolds

**Figure 1:**
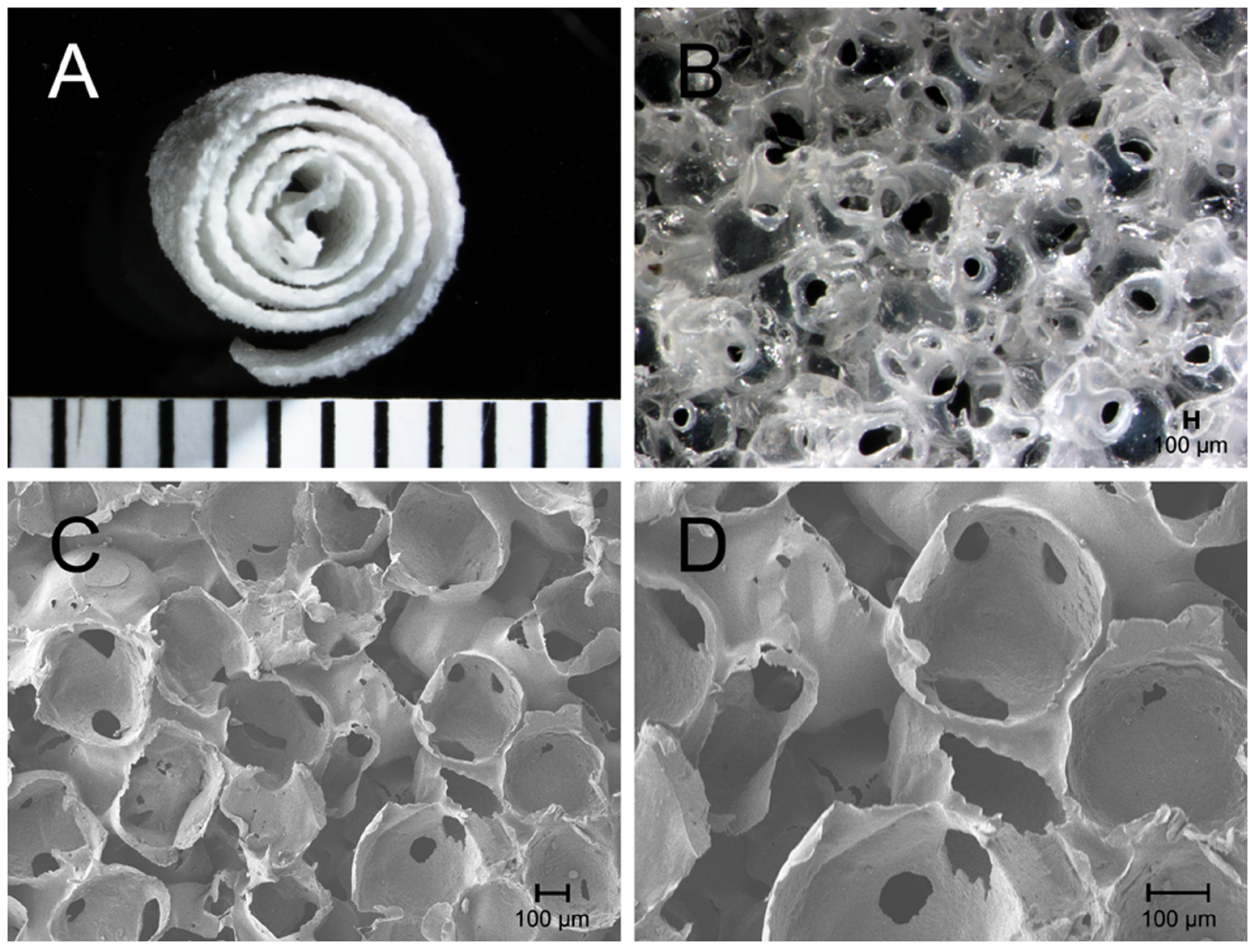
Representative photographs of nano-HA/PCL spiral scaffold. Gross view of the spiral scaffold (A). The morphology of nano-HA/PCL spiral scaffold under stereomicroscopy (B) and scanning electron microscopy (C and D).

A representative macrograph and micrograph of the obtained nano-HA/PCL spiral scaffold is depicted in Fig. 1. It can be seen from this figure that the scaffold was porous, and the pores were interconnected. This spiral scaffold contains two types of pores. One is the large pores created by the sale particles that had diameters ranging in size between 250 and 400 µm, and the other is the micropores on the surface of the spherical macropores. The diameter of micropore was measured to be on the average of several 10 s of micrometers. Furthermore, the inclusion of nano-HA powders had little effect on the changes in morphology with different weight proportions from 12.5 to 33.3% since pure PCL scaffolds had the same morphological structures (data not shown).

### 2. Cell viability on nano-HA/PCL spiral scaffolds

**Figure 2:**
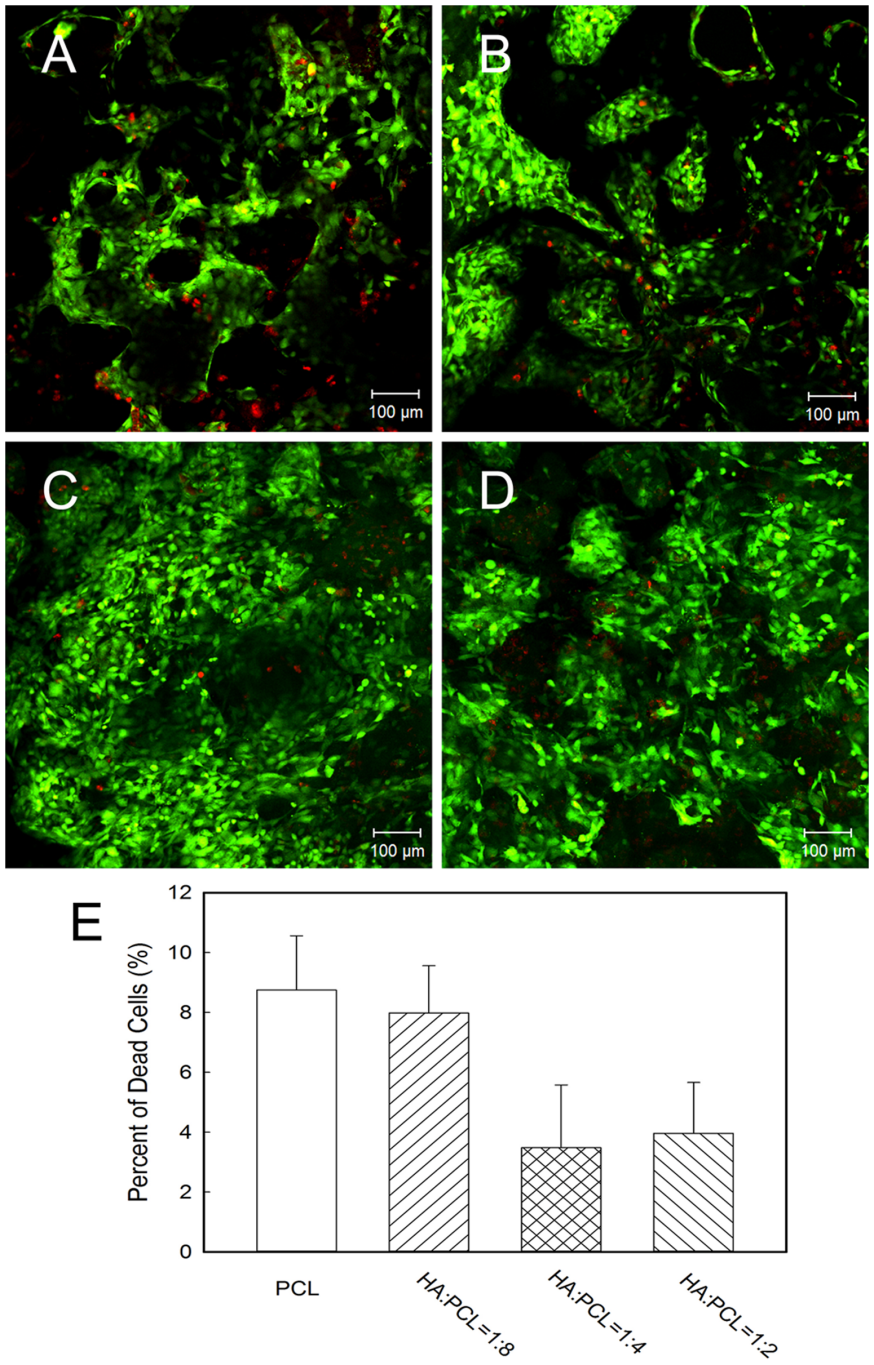
Cell viability cultured on nano-HA/PCL spiral scaffolds using Live/Dead assay. Cells cultured on PCL spiral scaffold (A), HA∶PCL = 1∶8 nano-HA/PCL spiral scaffold (B), HA∶PCL = 1∶4 nano-HA/PCL spiral scaffold (C), HA∶PCL = 1∶2 nano-HA/PCL spiral scaffold (D) for 7 days. (E) Quantitative analysis of the percent of dead cells within each spiral scaffolds. Live cells were stained green, dead cells were stained red and nucleus were stained blue. Data represent the mean ± standard deviation, n = 5.

Figure 2 showed nano-HA/PCL spiral scaffolds seeded with hFOBs after performing LIVE/DEAD staining. From the fluorescence signals it could be observed that cells adhered, proliferated, and remained viable after 7 days culture (Fig. 2 A–D). The very low red fluorescence depicted in the images indicated that there were a very low number of dead cells. However, more dead cells could be found in HA∶PCL = 1∶8 group and PCL group than in HA∶PCL = 1∶4 group and HA∶PCL = 1∶2 group (Fig. 2 E).

**Figure 3:**
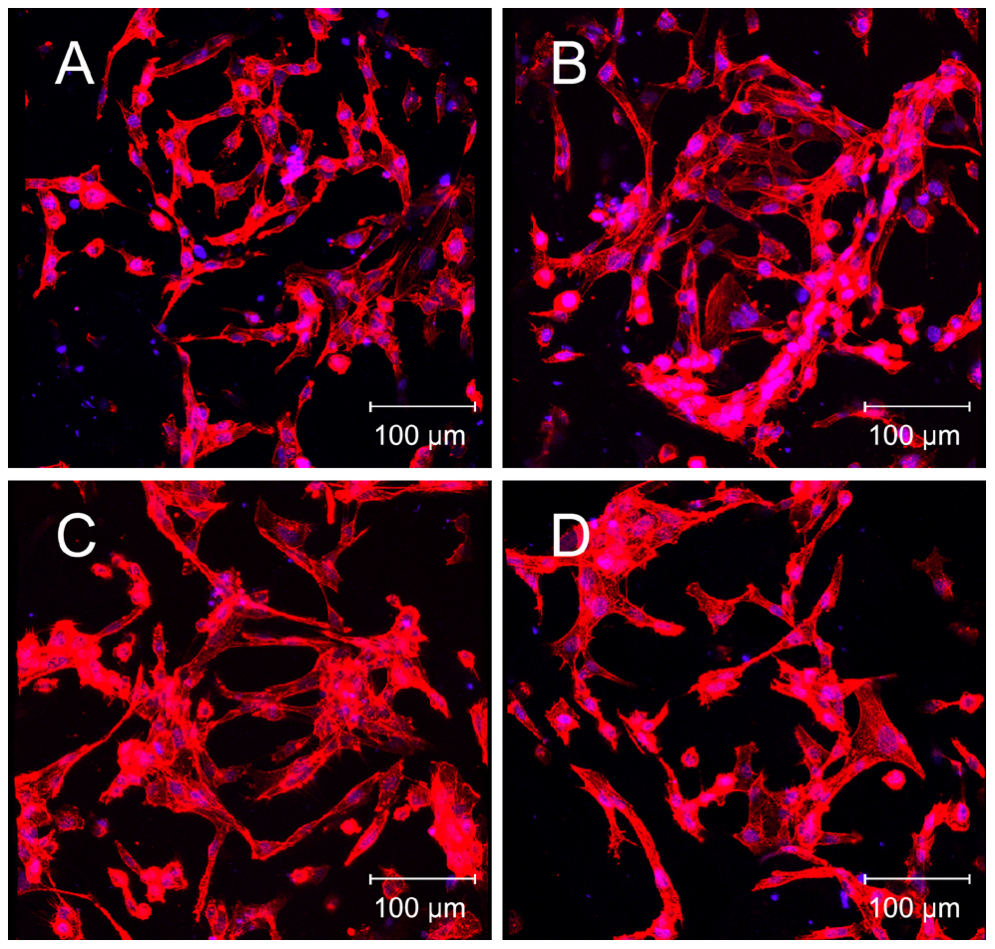
Cytoskeleton structure of osteoblast cultured on nano-HA/PCL spiral scaffolds for 4 days. F-actin staining of cells cultured on PCL spiral scaffold (A), HA∶PCL = 1∶8 nano-HA/PCL spiral scaffold (B), HA∶PCL = 1∶4 nano-HA/PCL spiral scaffold (C), HA∶PCL = 1∶2 nano-HA/PCL spiral scaffold (D). F-actin was stained red and nucleus was stained blue.

**Figure 4:**
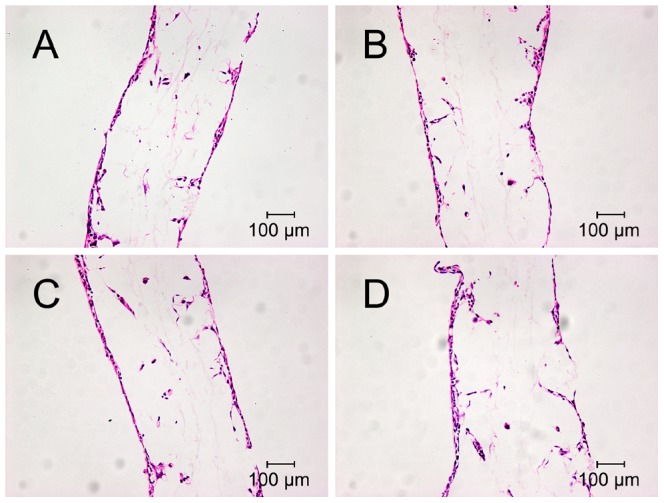
Cells morphology cultured on the nano-HA/PCL spiral scaffolds for 4 days by HE staining. Osteoblasts cultured on PCL spiral scaffold (A), HA∶PCL = 1∶8 nano-HA/PCL spiral scaffold (B), HA∶PCL = 1∶4 nano-HA/PCL spiral scaffold (C), HA∶PCL = 1∶2 nano-HA/PCL spiral scaffold (D).

The morphology of cells on the nano-HA/PCL spiral scaffolds was examined using fluorescence confocal microcopy and HE. After 4 days culture on spiral scaffolds, the cytoskeleton structure were examined using F-actin stain (Fig. 3). It can be observed that the morphology of hFOBs were similar in these four kinds of spiral scaffold. Abundant long actin stress fibers formed in cells were aligned along the interconnected pore of nano-HA/PCL spiral scaffolds. HE staining showed that lots of hFOBs were attached on the surfaces of nano-HA/PCL scaffolds. Furthermore, some cells were spread on the pore of scaffold (Fig. 4).

**Figure 5:**
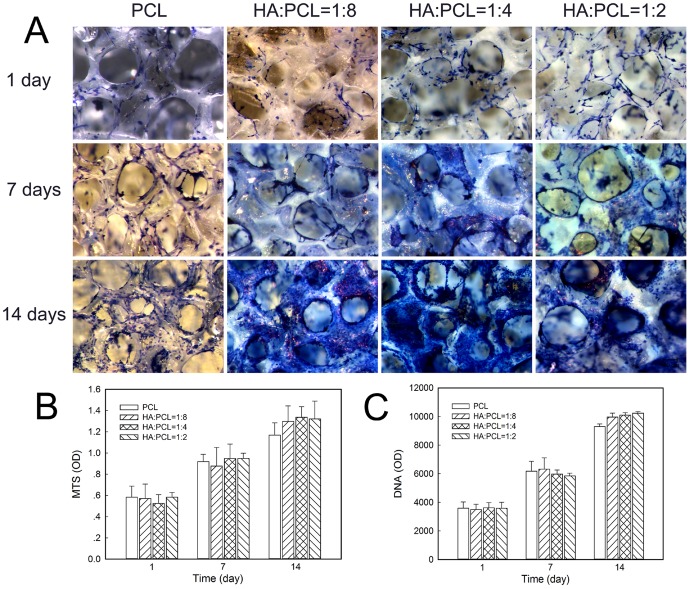
Osteoblasts distribution and proliferation cultured on the nano-HA/PCL spiral scaffolds after 1, 7, and 14 days culture. Cells distribution in the scaffolds evaluated by methylene blue staining (A). Quantitative analysis of cell proliferation measured by MTA assay (B) and PicoGreen DNA quantification assay (C). Data represent the mean ± standard deviation, n = 6.

To further investigate the cell adhesion and distribution within the nano-HA/PCL spiral scaffolds, hFOBs were cultured on the scaffolds for 1, 7, and 14 days and stained with methylene blue (Fig. 5 A). Staining showed cells were not only attached on the surfaces of scaffolds but also spread on the pore of scaffold at all indicated time endpoints. From the staining it can be seen that there were no differences in cellular attachment in four spiral scaffolds in each time group. With the increasing of culture time, the number of cells cultured in nano-HA/PCL spiral scaffolds was dramatically increased and cells were distributed throughout the spiral scaffolds. Furthermore, some cells were aligned together in the pore of scaffolds.

Proliferation of cells on nano-HA/PCLspiral scaffolds were analyzed using MTS assay and DNA assay. The data from the MTS assay (Fig. 5 B) showed that there were no significant differences in cell attachment in all spiral scaffolds at day 1. The results at day 14 indicate that cell proliferation occurred during this period in all the tested scaffolds, as indicated by the higher values of MTS at day 14 when compared to day 1 and day 7. However, the numbers of cells did not appear to make a statistically significant difference among the different ratio nano-HA/PCL spiral scaffolds and PCL spiral scaffolds. The results from the DNA assay were shown in Fig. 5 C. The DNA content on all spiral scaffolds increased gradually until day 14. Furthermore, there were still no significant differences in DNA content amongst the different ratio scaffolds at each time point.

### 3. Cell differentiation on nano-HA/PCL spiral scaffolds

**Figure 6:**
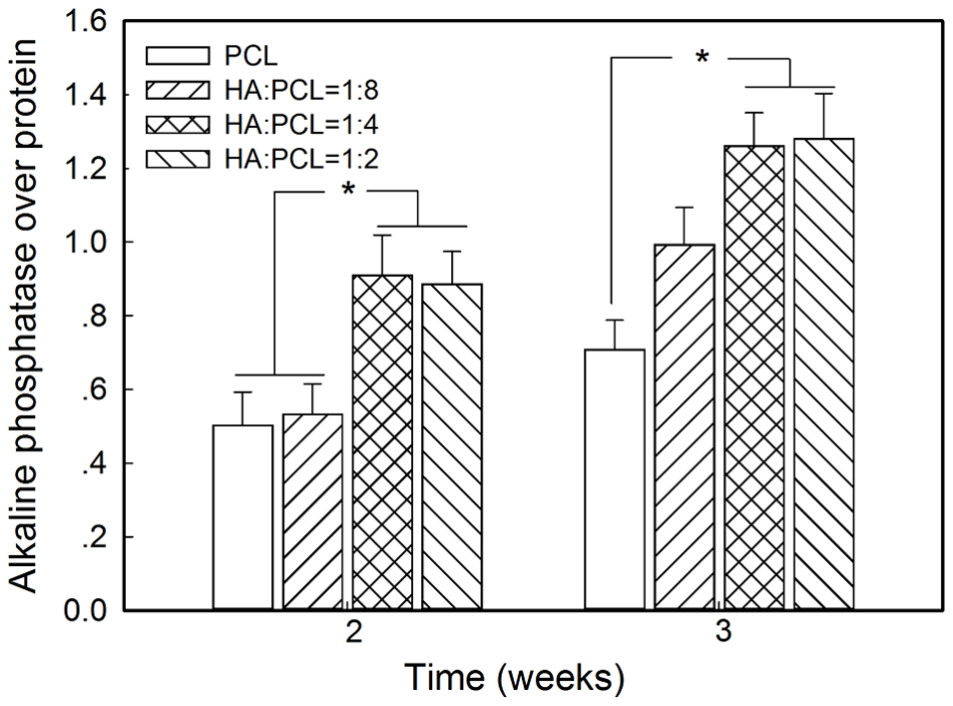
Alkaline phosphatase (ALP) expression on the nano-HA/PCL spiral scaffolds was normalized to protein concentration after 2 and 3 weeks of culture. Data represent the mean ± standard deviation, n = 6. Significant difference between different material groups were denoted as * (*p*<0.05).

ALP activity, normalized to protein concentration, was plotted in Fig. 6. After 2 weeks, the expression of ALP activity was significantly higher in the HA∶PCL = 1∶4 and HA∶PCL = 1∶2 spiral scaffolds compared with HA∶PCL = 1∶8 and PCL spiral scaffolds (*p*<0.05). While ALP expression increased in all tested spiral scaffolds after 3 weeks, ALP activity in the HA∶PCL = 1∶4 and HA∶PCL = 1∶2 spiral scaffolds continued to be expressed at significantly higher levels compared with those of PCL spiral scaffolds (*p*<0.05). However, no significant differences in the ALP expression were found between HA∶PCL = 1∶8 spiral scaffolds and HA∶PCL = 1∶4, or HA∶PCL = 1∶2 spiral scaffolds in 3 weeks. Furthermore, the levels of ALP expression in the HA∶PCL = 1∶4 spiral scaffolds were very similar to expression levels in the HA∶PCL = 1∶2 spiral scaffolds in 2 and 3 weeks.

**Figure 7:**
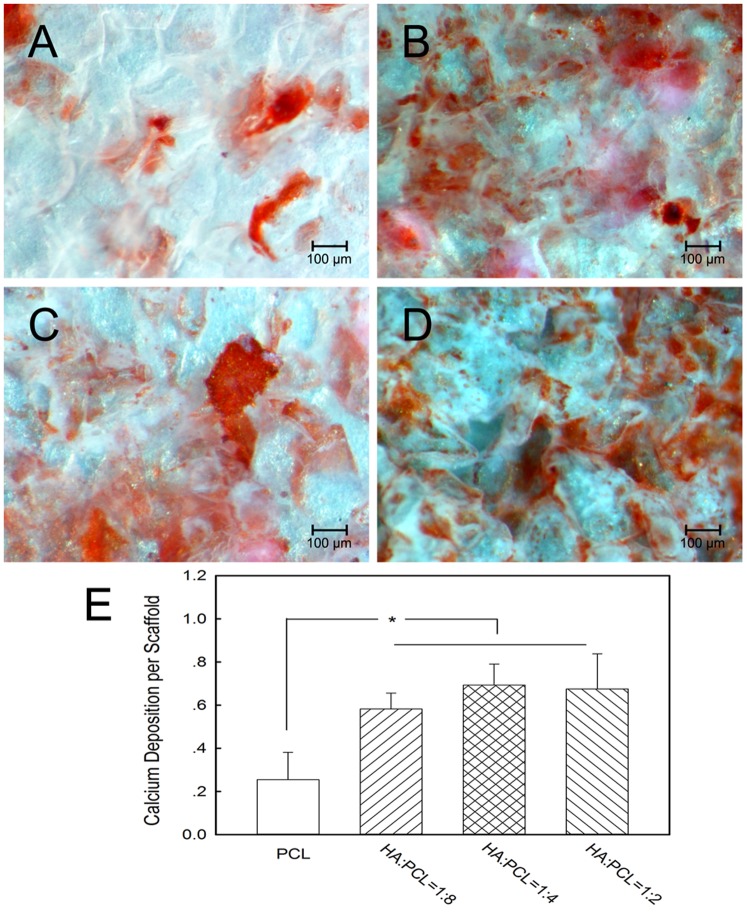
Alizarin S Red staining of calcium deposited on nano-HA/PCL spiral scaffolds. Cells cultured on PCL spiral scaffold (A), HA∶PCL = 1∶8 nano-HA/PCL spiral scaffold (B), HA∶PCL = 1∶4 nano-HA/PCL spiral scaffold (C), HA∶PCL = 1∶2 nano-HA/PCL spiral scaffold (D) for 21 days. (E) Quantitative analysis of the amount of calcium within each spiral scaffolds. Data represent the mean ± standard deviation, n = 6. Significant difference between different material groups were denoted as * (*p*<0.05).

Calcium assays were performed to assess the mineralized matrix formation on the scaffolds. It could be observed that a mineral matrix formed in the nano-HA/PCL spiral scaffolds (Fig. 7 A–D). Qualitative analysis showed that the average calcium deposition on nano-HA/PCL spiral scaffolds with HA content were significantly higher than that on PCL spiral scaffolds (*p*<0.05, Fig. 6E). The difference in calcium deposition was not statistically significant with varying HA content in the nano-HA/PCL spiral scaffolds (*p*>0.05). However, HA∶PCL = 1∶4 nano-HA/PCL spiral scaffolds had the highest amount of calcium accumulation.

**Figure 8:**
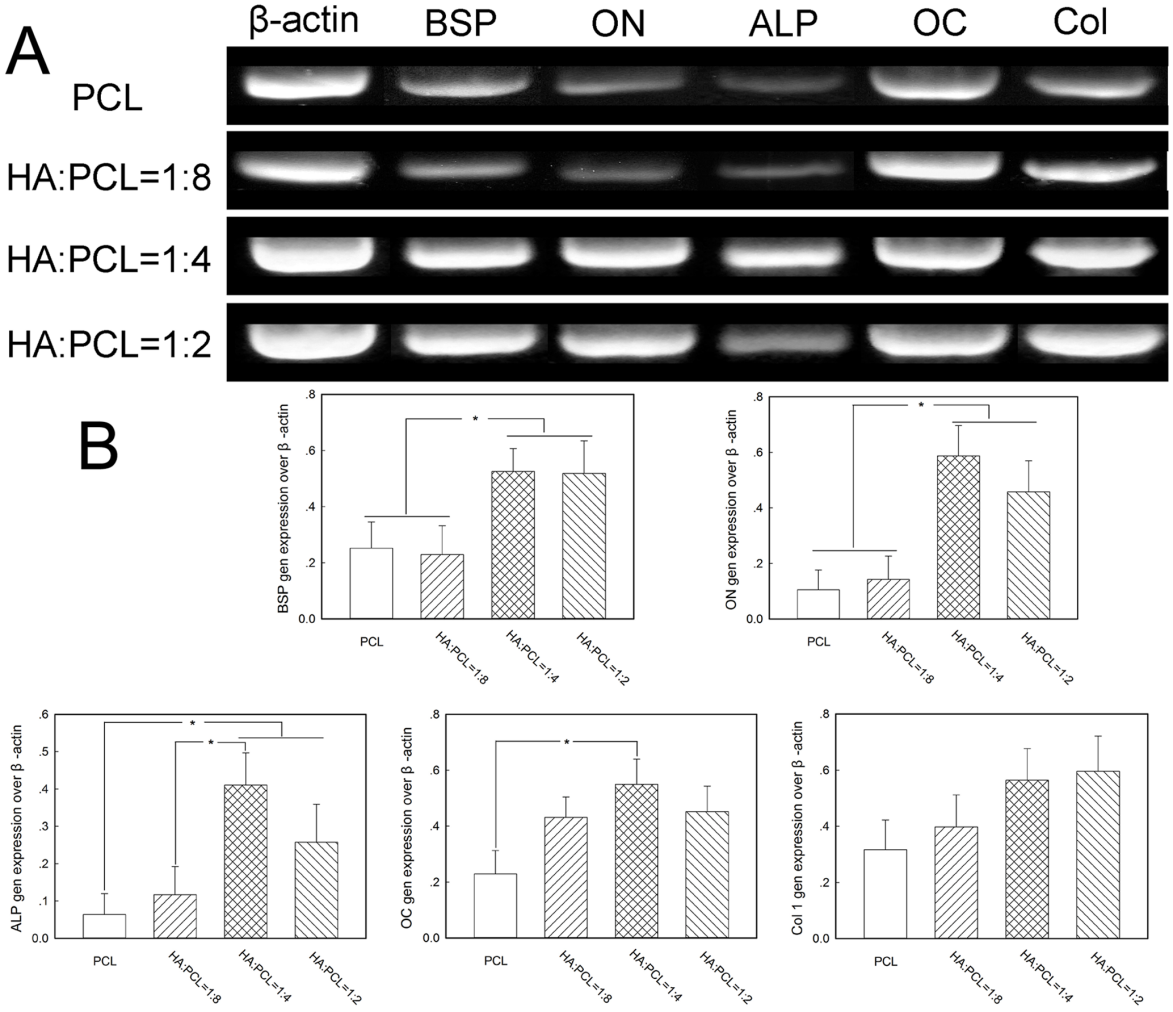
Gene expression of osteogenic markers in nano-HA/PCL spiral scaffolds after 3 weeks of culture. Representative electrophoresis gel (A) and semi-quantitative analysis of gene expression (B). The data presented were normalized with β-actin. Significant difference between different material groups were denoted as * (*p*<0.05).

We further examined the effect of nano-HA/PCL on osteoblastic cell differentiation at the mRNA level using a PCR technique (Fig. 8). After 3 weeks cultured on the nano-HA/PCL spiral scaffolds, BSP and ON mRNAs in the HA∶PCL = 1∶4 and HA∶PCL = 1∶2 spiral scaffolds were significantly higher than those in the HA∶PCL = 1∶8 and PCL spiral scaffolds (*p*<0.05). Interestingly, the levels of ALP mRNAs in the HA∶PCL = 1∶4 spiral scaffolds were the highest comparing with other three spiral scaffolds and were significantly higher than expression levels in the HA∶PCL = 1∶8 and PCL spiral scaffolds (*p*<0.05). The level of OC mRNAs in the PCL spiral scaffolds were significantly lower than expression levels in the HA∶PCL = 1∶4 spiral scaffolds (*p*<0.05). When examining levels of type I collagen in the nano-HA/PCL spiral scaffolds, Col mRNA expression in the nano-HA/PCL spiral scaffolds were the highest comparing to other bone differentiation related gen such as BSP, ON, ALP, and OC. However, there were no significant differences in the Col mRNA expression among all tested spiral scaffolds.

## Discussion

Bone tissue engineering is a potentially alternative strategy to repairing bone defects. A critical component of this tissue engineering approach is to develop an osteoconductive, porous, and biodegradable scaffold that may provide a temporary scaffold to guide new tissue in-growth and regeneration. Currently, biodegradable polymer/bioceramic composites have drawn considerable attention as a suitable scaffold material in bone tissue [12], [37]. This is because the hybridization of these materials can not only overcome the inflexibility and brittleness of hard ceramic materials, but also improve the osteoconductivity and degradation properties of polymers.

It is well known that the pore size and interconnectivity of scaffold are highly relevant to proper cell migration and proliferation as well as tissue vascularization and diffusion of nutrients and oxygen, which are necessary for bone formation. Previous studies also demonstrated that pore size between 100 and 350 µm is optimum for bone regeneration [38]. Interconnected porosity is also important for maximizing bone ingrowth leading to osteointegration and secure graft fixation. However, scaffolds produced by traditional salt leaching cannot guarantee interconnection of pores due to the low interconnectivity of particles randomly assembled in a polymer dissolved with a solvent [38], [39]. Furthermore, it is very difficult to control the pore size of scaffolds during evaporation and agglomeration of salt particles. In the present study, the porous nano-HA/PCL spiral scaffolds with different weight ratio of HA and PCL were fabricated by a modified salt leaching technique. From our SEM results, nano-HA/PCL spiral scaffolds fabricated with the modified salt leaching were composed of lots of macropores, which size was from 250 to 400 µm controlled by the salt size, and micropores in the range of about several 10 s of micrometers as well as formed interconnected porous structure. These microstructure including porosity, pore size and interconnection between pores indicate that nano-HA/PCL spiral scaffolds might be ideal scaffold as bone tissue engineering.

To investigate cell-scaffold interactions, hFOBs were seeded and cultured on the nano-HA/PCL spiral scaffolds. LIVE/DEAD staining showed that hFOBs maintained high cell viability in all spiral scaffolds when cells were cultured on the nano-HA/PCL spiral scaffolds. Moreover, it can be seen that cells were not only attached onto the surface of spiral scaffolds, but also distributed within the whole nano-HA/PCL spiral scaffolds from F-actin staining, HE staining, and methylene blue staining results. From the images it can be observed that the scaffolds with interconnected pores were instrumental in allowing the cells in penetrating throughout the entire scaffold. Considering that previous studies had demonstrated that cell infiltration and distribution within the whole scaffold will greatly affect the overall performance of the cells/scaffold construct, it can be seen that our porous scaffold offers an optimal biocompatible surface for bone regeneration [40]. Quantity analysis showed that the cell numbers were gradually increased with cultured time, but there were no significant difference in cell proliferation amongst the nano-HA/PCL spiral scaffolds with different weight of HA. This was in agreement with Shor et al. studies where they compared the cell activity between HA/PCL scaffolds contain with 25% weight HA and pure PCL scaffolds and Alamar blue assay showed no statistical difference between two groups [30]. Coincidentally, previous work had also reported that no significant difference between PCL and PCL/HA scaffolds fabricated by fused deposition modeling when using human calvarial osteoblast [31]. Contrarily to what was observed in other studies, porous HA/PCL scaffolds showed a significant increase in osteoblast adhesion and proliferation [22], [41]. In addition, Eosoly et al. examined the MC-3T3 mouse calvarial osteoblast cells response of different compositions of PCL/HA scaffolds (0, 15, and 30 weight % HA). Composites with lower HA content (15 weight %) showed the significantly higher cellular proliferation compared to that of higher HA content and pure PCL at the 7 and 14 days [29].

ALP, an early osteogenic marker for differentiation, is important for the construction of bone matrix. Our results showed that nano-HA/PCL scaffolds with higher HA content (HA∶PCL = 1∶4, 1∶2) had a significantly higher expression of ALP compared to scaffolds with lower HA content (HA∶PCL = 1∶8) or pure PCL scaffolds. Moreover, The release of ALP was the highest for cells grown in the HA∶PCL = 1∶4 scaffolds group at the different test point. This trend was confirmed by the mRNA expression of ALP after hFOBs were cultured on the nano-HA/PCL scaffolds for 3 weeks, which was reinforced semi-quantitatively by RT-PCR analysis. This observation was in agreement with another study, in which HA steadily increased ALP activity in PCL scaffolds containing with 25% weight HA from days 7 to 21 [30]. Furthermore, there was general consensus of our results with Ngiam et al.'s study whereby there was an enhancement of ALP activity on the PLGA/HA composite scaffolds [20]. Causa et al. examined the Saos-2 cell and human osteoblasts response of different compositions of HA/PCL composites (tested on composition of 13, 20, 32 volume % HA, namely 40, 70, and 132 weight % HA) fabricated by phase inversion and casting technique. The release of ALP was higher for cells grown in the HA/PCL scaffolds contain with 40% (w/w). Furthermore, human osteoblasts on these scaffolds contain with 132% (w/w) HA never reached the ALP levels measured for the other scaffolds [32]. Based on the above results, we can conclude that the ALP activity of osteoblast cultured on the HA/PCL scaffolds increased and then decreased with an increase in percentage of the HA in the HA/PCL scaffolds. Therefore, HA scaffolds induced osteoblastic cell differentiation in a dose dependent manner.

The mineralized matrix formation is a phenotypic marker for a later stage of osteogenic differentiation. The calcified matrix formation in the nano-HA/PCL spiral scaffolds was significantly elevated compared to that of pure PCL spiral scaffolds. Moreover, the formation of mineralized matrix was the highest for cells grown on the HA∶PCL = 1∶4 scaffolds group. These results were confirmed by the PCR results. The gene expression level of BSP, ON, OC, which are considered as late differentiation markers of the osteoblast phenotype, were significantly upregulated in hFOBs cultured on the nano-HA/PCL spiral scaffolds with higher HA content. All these differentiation markers had the highest expression in the HA∶PCL = 1∶4 spiral scaffolds group. Therefore, it was further demonstrated that HA scaffolds was capable of inducing cellular differentiation markers in a dose dependent manner. Previous studies also demonstrated that another biocermic, octacalcium phosphate, stimulates its own osteogenic capability in an octacalcium phosphate dose–dependent manner in vitro [42], [43].

## Conclusions

In the present study, we synthesized interconnected porous nano-HA/PCL scaffolds with different weight ratios of HA by a modified salt leaching technique. hFOBs can adhere, infiltrate the interconnected porous of the nano-HA/PCL spiral scaffolds, and also maintain high cellular viability. Although cell proliferation rate were unchanged when the amount of HA were changed in the nano-HA/PCL spiral scaffolds, ALP activity and mineralized matrix formation were significantly promoted with the HA content increase in the nano-HA/PCL spiral scaffolds. Moreover, the highest promotion occurred in the HA∶PCL = 1∶4 spiral scaffolds group, instead of lower or higher of HA group. The expression of osteogenic markers, such as ALP, BSP, ON, and OC, were also significantly upregulated in the HA∶PCL = 1∶4 spiral scaffolds. The results suggested the optimal blend of HA and PCL was around a 1 to 4 ratio by weight.

In future, more in-depth studies, including the investigation into the degradation, the mechanism of cell-nano-HA/PCL spiral scaffolds interaction, and in vivo osteoconductivity properties of these spiral scaffolds, are required to confirm the potential benefits of the nano-HA/PCL spiral scaffolds used for bone regeneration.
